# Editorial: Smart dust: Micro and nano scale devices for highly-integrated localized and distributed smart systems for precision and personalized medicine

**DOI:** 10.3389/fnbot.2022.1051124

**Published:** 2022-10-10

**Authors:** Paolo Motto Ros, Danilo Demarchi, Sandro Carrara

**Affiliations:** ^1^Department of Electronics and Telecommunications, Politecnico di Torino, Turin, Italy; ^2^Integrated Circuits Laboratory, École Polytechnique Fédérale de Lausanne, Neuchâtel, Switzerland

**Keywords:** biomedical CMOS circuits and systems, diagnostic telemetry, wireless power and data transfer, lab-on-CMOS, injectable micro-devices, biointerface engineering, micro-robot, magnetic control system

Wearable and implantable devices are nowadays a well-established research field, with application and devices out of the medicine field too, possibly part of our daily routines. From a historic perspective, the paved way is clear: from an overall macro monitoring and diagnostic approach, where only the effects of underlying processes are captured as a whole or indirectly, to a micro one, where biological and chemical processes are individually locally monitored (and eventually controlled). To this end, new bioelectronic devices (biochips) are needed: even if the idea of shrinking down to dust-sized devices has been conceived more than 20 years ago, the first real device was demonstrated in the body of a mammalian 6 years ago only, and many challenges are still there. It is not only a matter of simply reducing size, performances, figures-of-merit of (some) orders of magnitude while relying on already well-established designs and solutions, but new co-design paradigms, architectures, electronic circuits, bio-CMOS interfaces, nanosensors, have to be investigated and developed. However, designing, developing, and fabricating Smart Dust devices are just the first steps for effectively enabling a precision and personalized medicine at the micro and nano scale. In these initial (from an application perspective) steps, major focus and challenges are at the architectural/system/integration level of sensor/stimulation interfaces and circuits, communication and networking approaches, energy provisioning and management strategies. Broadly speaking, the emphasis is mostly in maximizing the efficiency, effectiveness, and reliability of the designed solution into achieving the specific goal, with a “device-centered” perspective (and often in a short-term timescale) (Motto Ros et al., 2017; Barbruni et al., 2020; Carrara, 2021).

Next steps in the development of Smart Dust applications should extend such perspective and take into consideration the impact on, and the interaction with, the surrounding (*in-vitro, ex-vivo, in-vivo*) biological environment (broadly speaking) of the micro and nano scale devices. With the papers published in this Research Topic we want to report some steps toward this direction.

In the paper “*Magnetically guided catheters, micro-and nanorobots for spinal cord stimulation*” Torlakcik et al. opportunities and challenges of magnetic navigation systems for steering the tethered devices are investigated in the context of Spinal Cord Stimulation (SCS). Starting from the current techniques in SCS lead placement and seeing magnetically controlled probes as precursors of untethered magnetic devices, the analysis is directed toward how small-scale devices could overcome complications and limitations, thus opening the way to improved (or new) standards of practice.

Magnetic control of micro-robots is the topic of the paper “*Study on magnetic control systems of micro-robots*” Shao et al. as well, but here the focus is on remote control systems of micro-robots in a 3D space with up to 8 degrees-of-freedom at a millimetric level. Pioneering works in this field are critically reviewed and categorized, in particular highlighting theoretical and practical pros and cons of stationary electromagnet, permanent magnet, and mobile electromagnet control systems. Finally, a new system is proposed and further challenges to be addressed in future works are outlined.

The paper “*Tracking the migration of injectable microdevices in the rodent brain using a 9.4 T magnetic resonance imaging scanner*” Khalifa et al. then moves a step forward in understanding the long-term behavior of implanted micro-devices by assessing their spatial stability under chronic conditions. The devices were precisely injected in six different rodent brain regions. Their position was accurately verified (although with some limitations) and monitored for up to 17 weeks. Histological analysis was then carried out to investigate the immunoreactivity around the micro-devices to better understand the anchoring/migration process. Even if the scar formation could negatively impact on the interface between the micro-device and the surrounding neural system, results show that only minimal drift occurred, thereby effectively opening the way for Smart Dust applications locally operating in the long-term.

With the same perspective, the paper “*Integrated micro-devices for a lab-in-organoid technology platform: current status and future perspectives*” Angotzi et al. aims at assessing the impact of an active silicon micro-device (fully working and targeting chronic recordings of neural activity) on the surrounding neural systems in a Lab-in-Organoid (LIO) context. Major challenges are power-related aspects; here concerns are focused on the biological effects of an inductive wireless power transfer as well. The fully-integrated CMOS design, fabrication, and characterization is discussed and then the impact of sinusoidal RF electromagnetic fields (2–6 GHz, used to power and communicate with the micro-device) is evaluated by means of immunofluorescence and MTT assay. Results show the feasibility and reliability of using specific time windows for wirelessly power and reading out data about the biological activity of the brain organoids, without (very likely) influencing the cellular viability; they overall confirm the feasibility of the proposed solution, both from an electronic engineering and a biological perspective, making it a significant step forward in the development of full Smart Dust applications.

## Author contributions

All authors listed have made a substantial, direct, and intellectual contribution to the work and approved it for publication.

## Conflict of interest

The authors declare that the research was conducted in the absence of any commercial or financial relationships that could be construed as a potential conflict of interest.

## Publisher's note

All claims expressed in this article are solely those of the authors and do not necessarily represent those of their affiliated organizations, or those of the publisher, the editors and the reviewers. Any product that may be evaluated in this article, or claim that may be made by its manufacturer, is not guaranteed or endorsed by the publisher.
